# Synthesis of Surfactants Derived from 2-Mercaptobenzimidazole and Study of Their Acute Toxicity and Analgesic and Psychotropic Activities

**DOI:** 10.1155/2019/9615728

**Published:** 2019-07-30

**Authors:** Terence Nguema Ongone, Latyfa El Ouasif, Mostafa El Ghoul, Redouane Achour, Hind Chakchak, Meryem El Jemli, Yahia Cherrah, Katim Alaoui, Amina Zellou

**Affiliations:** ^1^Laboratory of Organic Heterocyclic Chemistry, Competence Center Pharmacochemistry, Faculty of Science, Mohammed V University, Ibn Battuta Avenue BP 1014 RP, Rabat, Morocco; ^2^Laboratory of Pharmacology and Toxicology, Faculty of Medicine and Pharmacy, Mohammed V University, BP 6203 Rabat-Institutes, Rabat, Morocco; ^3^National Center for Scientific and Technical Research, Rabat, Morocco

## Abstract

The aim of the present study is to synthesize cationic salts from a relatively toxic compound named 2-mercaptobenzimidazole and to evaluate some of their pharmacological properties. The acute toxicity of these salts is evaluated according to OECD 423 Guidelines at the doses of 300 and 2000 mg/kg; their peripheral analgesic effect is studied using the Koster test at the therapeutic dose of 200 mg/kg and their sedative action is evaluated using Traction, Chimney, Hole-board, and Rotarod tests at the doses of 200 and 400 mg/kg. All synthesized molecules show no acute toxicity according to OECD Code 423 guidelines at doses ranging from 300 to 2000 mg/kg and do not cause any obesity or anorexia. Also, the results of the Koster test show that the studied compounds have an average analgesic effect at the dose of 200 mg/kg compared to acetylsalicylic acid. In addition, the elaborated compounds have shown a moderate sedative effect at the dose of 400 mg/kg, in comparison to 2-mercaptobenzimidazole (400 mg/kg) and Bromazepam (20 mg/kg). These compounds have no cataleptic and hypnotic effects on the central nervous system at the doses of 200 and 400 mg/kg. These results argue in favor of a possible integration of the most active salts tested in the pharmaceutical industry owing to their analgesic and sedative effects.

## 1. Introduction

Heterocyclic organic chemistry accounts for a large part of organic chemistry research conducted around the world. In particular, heterocyclic structures having one or more nitrogen atoms form the basis of many pharmaceutical, agrochemical, and veterinary products. Indeed, like nitrogenous bases that play a very important role in the human body, these molecules are a large part of the active ingredients of drugs marketed to date. We can, for example, mention the benzodiazepines marketed for their psychotropic effects on the central nervous system (Bromazepam, Diazepam, Clobazam, and Prazepam) and the benzimidazoles sold for their antihelminthic, antisecretory gastric, and antipsychotic effects (Mebendazole, Flubendazole, Timoprazole, Lanzoprazole, and Pimozide): these are biologically active nitrogenous heterocycles. The search for new benzimidazole molecules likely to have antimicrobial [1–3], anti-inflammatory [4–6], analgesic [7–9], antidiabetic [10], antioxidant [11–13], antitumor [14–16], antihistamines [17, 18], antihypertensives [19–21], antiviral [22–24], antifungal [25–27], and psychotropic [28–30] properties interesting remains current. The literature also reports a large body of work showing that benzimidazole derivatives are good corrosion inhibitors [31–33]. It is with this in mind that Latyfa El Ouasif et al. [34, 35] synthesized 2-mercaptobenzimidazole derivatives, in the form of dialkyl derivatives and surfactants, to study their antioxidant and antimicrobial activities and their corrosion inhibiting ability. Indeed, today, a large number of benzimidazole derivatives used as surfactants are reported in various studies [36–38]. Indeed, the long-chain benzimidazolium constitutes a very interesting family of cationic amphiphiles whose structure and the nature of the counterion can be easily modulated. This makes them particularly interesting for biological and especially pharmacological applications.

In order to valorize these molecules, we used another method of synthesis of surfactants derived from 2-mercaptobenzimidazole with potential surfactant properties in order to evaluate their acute oral toxicity and their analgesic and psychotropic properties on the nervous system.

## 2. Materials and Methods

### 2.1. Synthesis of 2-Mercaptobenzimidazole Derivatives

**Scheme 1:**
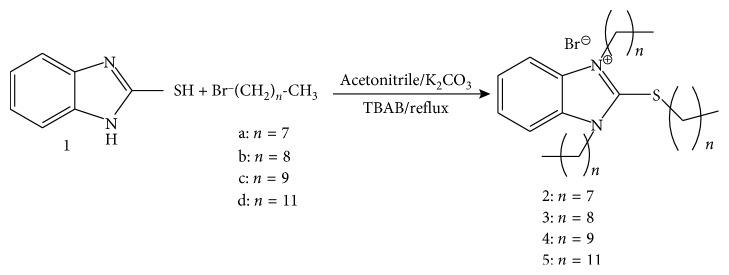
Synthesis of 1,3-dialkyl-2-alkylthio-1*H*-benzimidazolium bromides **2–5**.

In the works of Latyfa El Ouasif et al. previously mentioned, the surfactants **2–5** were prepared in two steps via dialkylated intermediates. However, in order to minimize material losses, we have used another method to synthesize these products in one step (Scheme 1). Thus, alkylation and quaternization of 2-mercaptobenzimidazole **1** with *n*-alkyl bromides was carried out under solid-liquid phase transfer catalysis conditions in the acetonitrile reflux in a single step. This method makes it possible to obtain 2-mercaptobenzimidazoliums bromides with good yields.

In addition, these molecules are prepared with the following method:

To a solution of 6.6 mmol of 2-mercaptobenzimidazole and 80 ml of acetonitrile was added 13 mmol of potassium carbonate, 0.66 mmol of tetra-n-butylammonium bromide and 20 mmol of 1-bromoalcane. The reaction mixture was refluxed for 72 hours. After evaporating, the solvent was removed under reduced pressure. The oil obtained is chromatographed on silica gel column (eluent: hexane).

The structures of the compounds are confirmed by ^1^H NMR, ^13^C NMR spectral data, and mass spectrometry (Table 1). The nuclear magnetic resonance spectra of the proton and carbon 13 were determined on an AVANCE 300 Bruker device at the 300 MHz frequency, in a solution of deuterated chloroform, and the mass spectra were recorded with a Q-TRAP device (Applied Biosystems) equipped with electrospray (ESI) sources.

### 2.2. Evaluation of Acute Toxicity

#### 2.2.1. Method Used

We conducted the acute toxicity study according to OECD Code 423 guidelines [39]. In this study, we used female mice, which are more sensitive than males. Their body weights were between 20 and 30 g. All these animals were healthy and were deprived of food for 12 hours before the start of the study.

In this study, the mice are placed in several groups of 3 animals. The control group received a solution of gum arabic (10%) orally. The other groups receive products **2–5** at the doses of 300 and 2,000 mg/kg, in two groups per product. For each dose, the test is performed twice to confirm the results.

We were particularly attentive to the behavior of the animals to note any signs of toxicity and mortality throughout the study period.

### 2.3. Peripheral Analgesic Activity: Koster Test

The study of the peripheral analgesic activity of our products was carried out using the Koster test [40], which involves inducing cramps in animals with acetic acid and observing the ability of our products to inhibit these cramps.

For this study, mice (females and males) with a body weight between 20 and 30 g were used. The animals are placed in several lots of 5 animals each. The control batch received an aqueous solution of 10% gum arabic. The reference batch received acetylsalicylic acid at the dose of 100 mg/kg. The other batches received different products studied at the dose of 200 mg/kg.

Cramps were induced by intraperitoneal injection of a 1.2% acetic acid solution at the rate of 0.15 ml for a 20 g mouse 60 minutes after administration of the tested products. The animals were placed in transparent cages, and the number of cramps was noted during a continuous observation of 10 min from the 5th minute after the injection of acetic acid. The percentage inhibition of abdominal cramps was calculated as follows:

(1) $$\begin{array}{c} \%\text{inhibition}=\left[\frac{\left(\text{Nt}-\text{Nc}\right)}{\text{Nt}}\right]\times 100, \end{array}$$

where Nt is the number of cramps in the control batch and Nc is the number of cramps in the treated batch.

### 2.4. Sedative Activity

The sedative activity of a given chemical is its ability to reduce or inhibit the level of alertness and psychomotor reactivity of the individual to whom it is administered. In rodents, it is studied through four behavior tests: Tractiont, Chimney, Hole-board, and Rotarod tests, in comparison with a drug.

#### 2.4.1. Traction Test

Traction test measures the time required for a mouse suspended by the forelegs to obtain traction of the hind limbs. The animals are placed on a small stretched wire (1 mm in diameter and 15 cm long) held firmly over the top of a bench with two wooden rods. Mice are allowed to grasp the wire with their forelegs and then released. A healthy mouse catches the wire with at least one of its hind legs in less than 5 seconds. Latency defects in animals receiving a product reflect a potential impairment of tensile force or poor coordination. This reflects the manifestation of sedation induced by a chemical [41, 42].

#### 2.4.2. Chimney Test

Chimney test is a simple test of tranquilization and muscle relaxation [43]. In this test, the animal is introduced into a 2000 mL glass tube head first. As soon as the mouse reaches the bottom of the tube on all fours, it tries to pull it up. The time elapsing between the moment when the mouse is placed on all fours and the moment when it tries to put back the test tube is recorded individually for each animal the time limit being 120 seconds. A normal mouse usually tries to escape in less than thirty seconds. Mice with a motor impairment are those who are unable to reassemble the test tube in less than 30 s. They will be said to be sedated [44, 45].

#### 2.4.3. Hole-Board Test

This test makes it possible to study the spontaneous locomotor activity for rodent exploration. The board we used measures 40 cm × 40 cm and was 2.2 cm thick. It has 16 holes 3 cm in diameter and is made of gray Perspex. The matte finish of the top panel prevents reflections that could alter the behavior of the animal. The mice were placed in the center of the perforated board, and they are let to explore freely for five minutes. The number of holes explored by each animal is carefully recorded over a five minute period [46, 47].

#### 2.4.4. Rotarod Test

Rotarod test is a standard test for assessing motor coordination, balance changes, and fatigue levels in rodents previously dosed with any product. This test is used to study the effects of different chemicals on the central nervous system [48, 49]. In this test, the mice were selected 24 hours before the test by retaining only those that could remain on the rotary bar (14 rpm) of the rotarod apparatus (Ugo Basile, Model 7600) for two consecutive periods of 60 seconds. The latency period before the fall of the animals is raised individually for each animal [50].

### 2.5. Hypnotic Activity

The hypnotic action of a substance is its ability to cause drowsiness or sleep. Animals receiving a dose of product are considered hypnotized if they lose their righting reflexes by putting them on their backs.

In this study, the mice received orally doses of 200 and 400 mg/kg of the products studied. The following items are recorded: the time of falling asleep (TE) and the time of sleep duration (TS) [51].

### 2.6. Cataleptic Activity

When it is possible to cross the anterior and posterior legs of the same side of animal that has received a dose of a product, it will be said that this product exerts a cataleptic effect on the animal [52]. In this study, we will note the moment when this effect arrives and the moment when it disappears.

### 2.7. Statistical Analysis

The results obtained in this work are expressed as mean values ± standard deviation (SD) for each data. These data were analyzed by one-way analysis of variance (one-way ANOVA). We used the post hoc procedure for significance of difference (*p* < 0.05 and *p* < 0.0001). The analysis was done with Graph pad Prism 6.0.

## 3. Results

### 3.1. Acute Toxicity Results

**Scheme 2:**
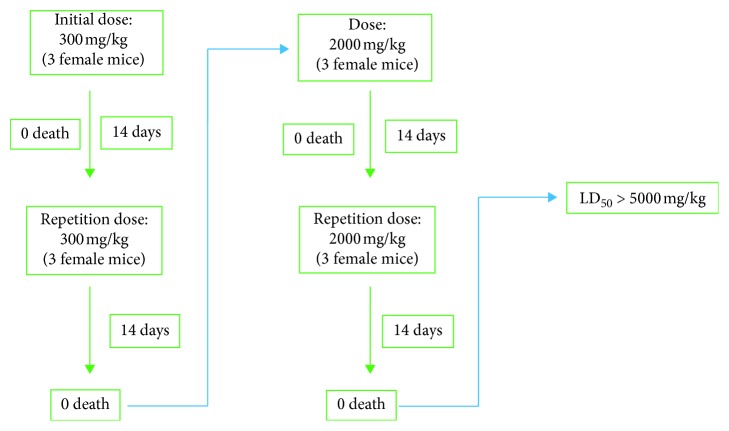
Determination of the LD_50_ of 1-alkyl-2-alkylthio-1*H*-benzimidazoles **2–5**.

All synthesized **2–5** molecules show no acute toxicity according to OECD Code 423 guidelines at doses ranging from 300 to 2000 mg/kg (Scheme 2). This could be related to the weight of these molecules, which would influence their bioavailability in the body and therefore the total dose acting on the animal. In addition, the weights of the treated animals remained relatively stable during the study for all products. Therefore, these compounds do not cause obesity or anorexia. It should also be noted that the study of the acute toxicity of 2-mercaptobenzimidazole, carried out as part of our previous work (work in progress), showed that its LD_50_ is as follows: LD_50_ = 1000 mg/kg.

### 3.2. Results of Peripheral Analgesic Activity

After careful study, the results obtained for this activity at the dose of 200 mg/kg vary according to the size of the molecule. Thus, 2-mercaptobenzimidazole **1** has a lower peripheral analgesic activity than acetylsalicylic acid at the therapeutic dose of 100 mg/kg. The number of cramps of this product is 26.02 ± 3.96 and that of acetylsalicylic acid is 9.60 ± 1.50. Moreover, the results obtained for the 1,3-dialkyl-2-alkylthio-1*H*-benzimidazolium bromides **2–5** show that the **3–5** derivatives are moderately active at this dose. They reduce with moderation the number of cramps induced by acetic acid in comparison with acetylsalicylic acid. Product **2** is weakly active at the same dose because it reduces slightly the number of cramps induced by acetic acid. The number of cramps recorded with these products is 25 ± 3.53 for product **2**, 19.60 ± 3.78 for product **3**, 17.60 ± 4.82 for product **4,** and 19.75 ± 4.24 for product **5** (Table 2).

### 3.3. Sedative Activity Results

#### 3.3.1. Traction Test Results

For this test, impaired motor coordination and impaired tensile force were observed in animals dosed with 400 mg/kg of 2-mercaptobenzimidazole **1** (Table 3). This is manifested by the falls of all the animals used with an average time of fall of 10.38 ± 0.14 s. This observation indicates that this product has a strong sedative effect at this dose on the central nervous system. On the other hand, this activity appears less important at the therapeutic dose of 200 mg/kg where we observe 33.33% of falls of the animals used with a average time of fall of 52.71 ± 30.42 s. Otherwise, there was a slight motor coordination deficiency and a slight alteration of the tensile strength of the animals receiving products **2–5** at the doses of 200 and 400 mg/kg (Tables 4 and 5). This is manifested by their recovery on the wire in less than 5 s. Thus, at the dose of 400 mg/kg, the straightening times of the animals are 2.36 ± 0.91 s for product **2**, 1.56 ± 0.60 s for product **3**, 1.51 ± 0.78 s for product **4,** and 1.20 ± 0.50 s for product **5**.

#### 3.3.2. Chimney Test Results

Administration of the therapeutic dose of 400 mg/kg of 2-mercaptobenzimidazole **1** significantly altered the motor coordination of the animals, as evidenced by the results of this test. This results, in particular, in the muscle relaxation observed since all the mice that have received this dose remain immobile at the bottom of the chimney (Table 3). This confirms the strong sedative effect of this product observed for the traction test. The results obtained at the therapeutic dose of 200 mg/kg show a less-significant sedative effect. Furthermore, the administration of 1,3-dialkyl-2-alkylthio-1*H*-benzimidazoliums bromides **2–5** at the therapeutic dose of 200 and 400 mg/kg orally does not modify the animal's motor coordination or their traction force. This is observed by the fact that mice take less than 30 s to search for the tube (Tables 4 and 5). Indeed, at the dose of 400 mg/kg, the average time taken by the animals to try to leave the test tube is 5.21 ± 1.33 s for the product **2**; 5.38 ± 2.06 s for product **3**; 4.82 ± 1.50 s for product **4;** and 6.61 ± 2.81 s for product **5**. These products exert a weak sedative effect on the central nervous system.

#### 3.3.3. Hole-Board Test Results

The results obtained with this test for 2-mercaptobenzimidazole **1** at the therapeutic dose 400 mg/kg show that the animals used lose their sense of curiosity since the number of holes explored is much lower than that of the control animals that have only received a solution of 10% gum arabic. In addition, this dose considerably decreases the spontaneous locomotor activity of animals because the space traveled between the holes is very small (Table 3). These results confirm a strong sedative effect observed in previous tests. We found, however, that the administration of 1,3-dialkyl-2-alkylthio-1*H*-benzimidazoliums bromides **2–5** at therapeutic doses of 200 and 400 mg/kg resulted in a slight decrease in the cumulative number of holes explored by the animals (in relation curiosity) and the number of spaces traveled between two holes (in relation to the motor activity). They therefore retain their spontaneous locomotor activity (Tables 4 and 5). Indeed, at the dose of 400 mg/kg, the average number of holes explored by the animals is 3.96 ± 1.95 holes for the product **2**; 3.60 ± 0.67 holes for product **3**; 3.88 ± 1.51 holes for product **4;** and 4.72 ± 1.40 holes for product **5**. These results show that these products exert a slight sedative effect on the central nervous system.

#### 3.3.4. Rotarod Test Results

The results obtained with this test show that all the animals having received the dose of 400 mg/kg of 2-mercaptobenzimidazole **1** completely lose their motor coordination and their equilibrium on the rotating stem. They also show signs of tiredness due to the effect of the product (Table 3). This confirms the strong sedative effect of the product **1** on the central nervous system. The results obtained with of 1,3-dialkyl-2-alkylthio-1*H*-benzimidazoliums bromides **2–5** show that all the animals which have received the therapeutic doses of 200 and 400 mg/kg effectively preserve their motor coordination and their equilibrium on the rotating stem in comparison with the control animals having received only a solution of 10% gum arabic (Tables 4 and 5). Indeed, these animals put more than 120 s on the rotating stem as the control animals. In addition, these animals do not exhibit particular fatigue effects. This result confirms the weak sedative effect of these molecules on the central nervous system.

### 3.4. Hypnotic Activity

The products tested do not exert hypnotic effects on the central nervous system at the doses of 200 and 400 mg/kg.

### 3.5. Cataleptic Activity

The products tested do not exert cataleptic effects on the central nervous system at the doses of 200 and 400 mg/kg.

## 4. Discussion

In order to carry out antibacterial and antioxdant activities, El Ouasif et al. [34] synthesized 1,3-dialkyl-2-alkylthio-1*H*-benzimidazolium bromides following two steps. The first corresponds to the synthesis of 1-alkyl-2-alkylthio-1*H*-benzimidazole under phase transfer catalysis conditions and the second concerns the quaternization of 1-alkyl-2-alkylthio-1*H*-benzimidazole at reflux of acetonitrile. In this context, the present work aims to reprepare these products owing to their pharmacological interest and to valorize them by studying in vivo their acute toxicity, their peripheral analgesic activity, and their psychotropic effects on the central nervous system. For this purpose, we have carried out the alkylation of 2-mercaptobenzimidazole and the quaternization of 1-alkyl-2-alkylthio-1*H*-benzimidazole in a single step under solid-liquid phase transfer catalysis conditions in the acetonitrile reflux (Scheme 1). The acute toxicity evaluation of products **2–5** at the dose of 300 mg/kg induced no signs of toxicity or mortality. Also, the toxicity at the dose of 2000 mg/kg was evaluated twice without observing any death or any sign of toxicity during the entire observation period. In addition, no significant change in the weight of the treated animals was noted. These results show that the tested salts **2–5** are not toxic at 300 and 2000 mg/kg. Their respective LD_50_ is greater than 5000 mg/kg (Scheme 2). Therefore, they belong to the category V of the Globally Harmonized System (GHS). These results corroborate those of our previous work in which we have shown that the insertion of long alkyl chains on 4-phenyl-1,5-benzodiazepin-2-one leads to nontoxic products [53]. The literature also mentions a large number of studies showing the absence of toxicity on molecules with long carbon chains [54–56]. However, a chronic toxicity study would be required to complete our study. Furthermore, we studied the peripheral analgesic activity of products **1** and **2–5** at the therapeutic dose of 200 mg/kg which consists on evaluating the ability of these products to inhibit the cramps induced in mice by 1.2% acetic acid. The number of cramps was noted for ten minutes from the fifth minute after the injection of acetic acid to animals that received the products studied an hour earlier. The obtained results show that 2-mercaptobenzimidazole **1** and 1,3-dioctyl-2-octylthio-1*H*-benzimidazolium bromide **2** are weakly active, whereas 1,3-di(nonyl decyl, dodecyl)-2-[(nonyl, decyl, dodecyl)thio]-1*H*-benzimidazolium bromides **3–5** are moderately active. Indeed, these molecules have a cramp reduction percentage of 33.67 ± 10.03% for the product **1**; 36.70 ± 8.95% for product **2**; 50.37 ± 9.57% for product **3**; 55.44 ± 12.22% for product **4;** and 50 ± 10.81% for product **5**. This analgesic effect can be explained by a possible involvement of opioid receptors [57]. These results are in agreement with the work of Kuzmierkiewiez et al. [58] and those of Taniguchi et al. [59], which showed that certain 3-(benzimidazol-2-yl)propanoic acid and 2-aminobenzimidazole derivatives have an analgesic effect. In addition, Merck and Dohme [60] reported that thiabendazole also has analgesic effects. In addition, in our recent work [61], we have shown that the long-chain carbon derivatives of 4-phenyl-1,5-benzodiazepin-2-one have a good analgesic effect on the nervous system. However, knowing that analgesic activity involving opioid receptors is more important in the case of small molecules, we can suggest first, that their analgesic effects may not be due to the involvement of the opioid system [62]. In addition, knowing that the transport of products **2–5** via specific receptors requires an affinity between the ligands (here molecules **2–5**) and the receptor, we can suggest that this affinity would be favored by the electrostatic forces of the alkyl chains, which would increase their analgesic action. Third, since these products are administered orally, they can be metabolized, and their potential metabolites may be the cause of their activity. Finally, this activity could be explained by the presence of the counterion (Br^−^) electron donor in the molecules **2–5**. Indeed, this hypothesis would be consistent with a series of studies [63–65], which show that the presence of an electron donor group in a molecule reinforces its lipophilic character that can in turn be responsible for the analgesic activity. Moreover, we have shown that all the studied products did not exert cataleptic and a hypnotic effects on the central nervous system at the therapeutic doses of 200 and 400 mg/kg, whereas the product **1** has a strong sedative effect, as it is shown by the different results of the behavioral tests. Indeed, we showed that this product caused the fall of 100% of the animals used in the traction test at the dose of 400 mg/kg (Table 3). These falls of animals reflect the alteration of their traction force and their motor coordination. The test of the chimney shows a relaxation of the muscles of the animals at this dose, since the latter remain more than 120 s in the test tube without trying to get out while the control animals remain just 3.40 ± 0.5 s and then try to escape. These observations corroborate as the results of the hole-board test where we observe a loss of locomotor activity in animals. Indeed, the animals having received 400 mg/kg of the product **1** explore on average 2.52 ± 1.03 holes, whereas the control animals explore on average 7 ± 1 holes. Finally, we observe a loss of equilibrium on the rotating rod in the rotarod test. These results are in agreement with those of Maryanoff et al. who showed that pyrido[1,2-a]benzimidazole derivatives [66] exert psychotropic effects on the central nervous system. However, products **2–5** have a weak sedative effect on the central nervous system. Indeed, Traction test shows that the animals having received the dose of 400 mg/kg from these products retain their motor coordination. Animals hooked on the wire put on average 2.36 ± 0.91 s to recover completely when they received the product **2**. This time is 1.56 ± 0.60 s for the animals received the product **3**, 1.51 ± 0.78 s for those received product **4,** and 1.20 ± 0.50 s for those received product **5** (Tables 4 and 5). Similarly, Chimney test shows the absence of muscle relaxation for the animals that have received the different elaborated salts since these animals put less than 7 s to try to leave the test tube. The animals that received the product **2** put on average 5.21 ± 1.33 s trying to escape, those who received the product **3** put on average 5.38 ± 2.06 s, those who received the product **4** put on average 4.82 ± 1.50 s, and those who received the product **5** put on average 6.61 ± 2.81 s to leave the test tube (Tables 4 and 5). The results of these two tests are supported by those of the Hole-board test, which show that animals received product **2** explore on average 3.96 ± 1.95 holes, the animals having received the product **3** explore on average 3.60 ± 0.67, those who received the product **4** explore an average of 3.88 ± 1.51, and the mice having received the product **5** explore on average 4.72 ± 1.40 holes (Tables 4 and 5). These results show that the locomotor activity of the animals receiving the prepared salts is slightly impaired. The results of the Rotarod test confirm this low sedative activity of products **2–5** at the dose of 400 mg/kg since the animals receiving them remain more than 120 s (Tables 4 and 5) on the rotating stem, thus maintaining their equilibrium. Our results are consistent with the work of Sercdcnin and Yarkova [67], who have shown that some benzimidazoles can be active in the central nervous system without having a sedative effect. Finally, we do not notice any significant change in results as we move from one derivative to another and from one dose to another in the case of products **2–5**. In view of the good results obtained in this work, we suggest the use of 2-mercaptobenzimidazole **1** for its sedative effect and 1,3-di(nonyl, decyl, dodecyl)-2-[(nonyl, decyl, dodecyl)thio]-1*H*-benzimidazolium bromides **3–5** for their analgesic activity in the pharmaceutical industry. However, the use of these molecules as drugs requires that they should be soluble in the body, to ensure their transport, their diffusion, and better cell penetration. In addition, the determination of the molecular targets on which these molecules act would be very important to know their molecular mechanisms. Finally, we suggest studying the anticonvulsive, antihelminthic, antigastric secretory, antihistaminic, and anti-inflammatory activity of these products in order to display their numerous pharmacological benefits.

## 5. Conclusion

The cationic salts derived from 2-mercaptobenzimidazole prepared in the present study are not toxic at the doses of 300 and 2000 mg/kg and do not cause any obesity or anorexia. Therefore, they belong to the category V of the Globally Harmonized System (GHS). Furthermore, our results show that these salts have an average peripheral analgesic effect at the dose of 200 mg/kg compared to acetylsalicylic acid (100 mg/kg).

In addition, they do not have cataleptic and hypnotic effects on the central nervous system at the doses of 200 and 400 mg/kg, but have a weak sedative effect at the dose of 400 mg/kg compared to Bromazepam (20 mg/kg). 2-mercaptobenzimidazole has a strong sedative action comparable to that of Bromazepam (20 mg/kg) at the dose of 400 mg/kg and a low peripheral analgesic effect at the dose of 200 mg/kg.

## Figures and Tables

**Table 1:** Spectral data ^1^H NMR, ^13^C NMR, and mass spectrometry.

Products	^1^H NMR spectrum (*δ* in ppm and *J* in Hz)	^13^C NMR spectrum (*δ* in ppm and *J* in Hz)	Mass spectrum M^+^ (m/z)	Yields (%)
1,3-Dioctyl-2-(octylthio)-1*H*-benzimidazolium bromide **2**	0.85 (6H, m, CH_3_); 1.24–1.86 (36H, m, CH_2_); 3.38 (t, 2H, SCH_2_, *J* = 13.8); 4.29 (t, 2H, NCH_2_, *J* = 15.3); 7.18–7.26 (4H, m, H Ar)	14.1 (CH_3_); 22.6–32.9 (CH_2_); 34.1 (SCH_2_); 44.8(NCH_2_); 109.1–132.1 (C Ar); 168.9 (C=N)	487	90

1,3-Dinonyl-2-(nonylthio)-1*H*-benzimidazolium bromide **3**	0.85 (6H, m, CH_3_); 1.24–1.86 (36H, m, CH_2_); 3.38 (t, 2H, SCH_2_, *J* = 13.8); 4.29 (t, 2H, NCH_2_, *J* = 15.0); 7.17–7.26 (4H, m, H Ar)	14.1 (CH_3_); 22.6–32.9 (CH_2_); 34.0 (SCH_2_); 44.8 (NCH_2_); 109.0–132.1 (C Ar); 169.0 (C=N)	529	85

1,3-Didecyl-2-(decylthio)-1*H*-benzimidazolium bromide **4**	0.84 (6H, m, CH_3_); 1.22–1.84 (36H, m, CH_2_); 3.36 (t, 2H, SCH_2_, *J* = 13.8); 4.27 (t, 2H, NCH_2_, *J* = 15.3); 7.16–7.40 (4H, m, H Ar)	14.1 (CH_3_); 22.7–32.8 (CH_2_); 33.9 (SCH_2_); 44.8 (NCH_2_); 108.9–132.1 (C Ar); 169.1 (C=N)	571	82

1,3-Didodecyl-2-(dodecylthio)-1*H*-benzimidazolium bromide **5**	0.87 (6H, m, CH_3_); 1.25.1.86 (36H, m, CH_2_); 3.38 (t, 2H, SCH_2_, *J* = 13.8); 4.29 (t, 2H, NCH_2_, *J* = 15.0); 7.17–7.26 (4H, m, H Ar)	14.1 (CH_3_); 22.7–32.9 (CH_2_); 33.8 (SCH_2_); 44.9 (NCH_2_); 108.9–132.1 (C Ar); 169.1 (C=N)	655	74

**Table 2:** Effect of 2-mercaptobenzimidazole **1** and derivatives **2–5** on acetic acid-induced cramps in mice.

Products	Doses (mg/kg)	Number of cramps in 10 min	Percentage of inhibition (%)
**1**	200	26.20 ± 3.96^*∗*^	33.67 ± 10.03^*∗*^
**2**	200	25.00 ± 3.53^*∗*^	36.70 ± 08.95^*∗*^
**3**	200	19.60 ± 3.78^*∗*^	50.37 ± 09.57^*∗*^
**4**	200	17.60 ± 4.82^*∗*^	55.44 ± 12.22^*∗*^
**5**	200	19.75 ± 4.24^*∗*^	50.00 ± 10.81^*∗*^
Acetylsalicylic acid	100	09.60 ± 1.50^*∗∗*^	75.69 ± 03.79^*∗∗*^
Control	—	39.50 ± 2.60	—

^*∗*^ *p* < 0.05; ^*∗∗*^*p* < 0.0001 compared to control.

**Table 3:** Results of the sedative activity of 2-mercaptobenzimidazole at the doses of 200 and 400 mg/kg.

		Witness	Bromazepam	2-Mercaptobenzimidazole (200 mg/kg)	2-Mercaptobenzimidazole (400/kg)
Traction test	Percentage of falls (%)	0	100^*∗∗*^	33.33^*∗*^	100^*∗∗*^
	Average fall time (s)	0	10.0 ± 0.9^*∗*^	52.71 ± 30.42^*∗∗*^	10.38 ± 0.14^*∗*^
	Average recovery time (s)	0.5 ± 0.1	0	33.08 ± 3.74^*∗∗*^	0
Chemney test	Average time to climb the chemney (s)	3.40 ± 0.5	>120	>120	>120
Hole-board test	Number of holes explored over 5 min	7 ± 1	0^*∗∗*^	2.52 ± 1.03^*∗∗*^	1.68 ± 0.99^*∗∗*^
Rotarod test	Time spent on stem (s)	120 ± 0	1.2 ± 0.9^*∗∗*^	10.80 ± 1.84^*∗∗*^	1.60 ± 0.43^*∗∗*^

^*∗*^ *p* < 0.05; ^*∗∗*^*p* < 0.0001.

**Table 4:** Results of the sedative activity of the products **2** and **3** at the doses of 200 and 400 mg/kg.

		Witness	Bromazepam	**2** (200 mg/kg)	**2** (400 mg/kg)	**3** (200 mg/kg)	**3** (400 mg/kg)
Traction test	Percentage of falls (%)	0	100^*∗∗*^	0	0	0	0
	Average fall time (s)	0	10.0 ± 0.9^*∗*^	0	0	0	0
	Average recovery time (s)	0.5 ± 0.1	0	1.32 ± 0.49	2.36 ± 0.91^*∗*^	1.14 ± 0.17	1.56 ± 0.60
Chemney test	Average time to climb the chemney (s)	3.40 ± 0.5	>120	7.95 ± 3.41^*∗∗*^	5.21 ± 1.33^*∗*^	1.08 ± 0.25^*∗*^	5.38 ± 2.06^*∗∗*^
Hole-board test	Number of holes explored over 5 min	7 ± 1	0^*∗∗*^	4.84 ± 1.57	3.96 ± 1.95^*∗*^	5.12 ± 1.40	3.60 ± 0.67^*∗*^
Rotarod test	Time spent on stem (s)	120 ± 0	1.2 ± 0.9^*∗∗*^	120 ± 0	120 ± 0	120 ± 0	120 ± 0

^*∗*^ *p* < 0.05; ^*∗∗*^*p* < 0.0001.

**Table 5:** Results of the sedative activity of the products **4** and **5** at the doses 200 and 400 mg/kg.

		Witness	Bromazepam	**4** (200 mg/kg)	**4** (400 mg/kg)	**5** (200 mg/kg)	**5** (400 mg/kg)
Traction test	Percentage of falls (%)	0	100^*∗∗*^	0	0	0	0
	Average fall time (s)	0	10.0 ± 0.9^*∗*^	0	0	0	0
	Average recovery time (s)	0.5 ± 0.1	0	1.08 ± 0.25	1.51 ± 0.78	1.66 ± 0.18^*∗*^	1.20 ± 0.50
Chemney test	Average time to climb the chemney (s)	3.40 ± 0.5	>120	5.17 ± 1.71	4.82 ± 1.50	3.76 ± 1.31	6.61 ± 2.81^*∗∗*^
Hole-board test	Number of holes explored over 5 min	7 ± 1	0^*∗∗*^	5.36 ± 0.92	3.88 ± 1.51^*∗*^	4.88 ± 1.30	4.72 ± 1.40^*∗*^
Rotarod test	Time spent on stem (s)	120 ± 0	1.2 ± 0.9^*∗∗*^	120 ± 0	120 ± 0	120 ± 0	120 ± 0

^*∗*^ *p* < 0.05; ^*∗∗*^*p* < 0.0001.

## Data Availability

The data used to support the findings of this study are included within the article.
